# PROMIS — Prostate MR imaging study: A paired validating cohort study evaluating the role of multi-parametric MRI in men with clinical suspicion of prostate cancer^☆^

**DOI:** 10.1016/j.cct.2015.02.008

**Published:** 2015-05

**Authors:** A. El-Shater Bosaily, C. Parker, L.C. Brown, R. Gabe, R.G. Hindley, R. Kaplan, M. Emberton, H.U. Ahmed, on behalf of the PROMIS Group

**Affiliations:** a Division of Surgery and Interventional Science, University College London, UK; b Department of Urology, UCLH NHS Foundation Trust, UK; c Department of Academic Urology, Royal Marsden Hospital, Sutton, UK; d MRC Clinical Trials Unit at UCL, UK; e Department of Health Sciences, University of York, UK; f Department of Urology, Hampshire Hospitals NHS Foundation Trust, UK

**Keywords:** Prostate cancer, Transrectal ultrasound guided biopsy, Template transperineal mapping biopsy, Magnetic resonance imaging, Multi-parametric MRI, Triage diagnostic test

## Abstract

**Background:**

Transrectal ultrasound-guided prostate biopsies are prone to detection errors. Multi-parametric MRI (MP-MRI) may improve the diagnostic pathway.

**Methods:**

PROMIS is a prospective validating paired-cohort study that meets criteria for level 1 evidence in diagnostic test evaluation. PROMIS will investigate whether multi-parametric (MP)-MRI can discriminate between men with and without clinically-significant prostate cancer who are at risk prior to first biopsy. Up to 714 men will have MP-MRI (index), 10–12 core TRUS-biopsy (standard) and 5 mm transperineal template mapping (TPM) biopsies (reference). The conduct and reporting of each test will be blinded to the others.

**Results:**

PROMIS will measure and compare sensitivity, specificity, and positive and negative predictive values of both MP-MRI and TRUS-biopsy against TPM biopsies. The MP-MRI results will be used to determine the proportion of men who could safely avoid biopsy without compromising detection of clinically-significant cancers. For the primary outcome, significant cancer on TPM is defined as Gleason grade >/= 4 + 3 and/or maximum cancer core length of ≥ 6 mm. PROMIS will also assess inter-observer variability among radiologists among other secondary outcomes. Cost-effectiveness of MP-MRI prior to biopsy will also be evaluated.

**Conclusions:**

PROMIS will determine whether MP-MRI of the prostate prior to first biopsy improves the detection accuracy of clinically-significant cancer.

## 1 Background & introduction

Prostate cancer is the most common male cancer, with a doubling in incidence over the last 15 years in the UK. Currently over 40,000 new cases are diagnosed every year in the UK [1,2] and 223,307 new cases in the USA [3]. Many prostate cancers currently detected are clinically insignificant and do not have any clinical impact on the individual during his remaining life if left untreated [4,5]. This assertion has received considerable support from a number of large randomised controlled trials of prostate cancer screening and treatment. Whilst the PLCO (Prostate, Lung, Colon and Ovaries) screening trial in the USA showed no evidence of a survival benefit it was criticised for significant contamination (i.e., PSA testing) in the control arm [6]. The European Randomized Study of Screening for Prostate Cancer (ERSPC) showed a modest reduction in risk of death from prostate cancer in those screened [7]. The number needed to invite for screening was 936 and the number of prostate cancers needed to detect and/or treat was 33 to extend the life of one man over a 12 year period [8–10]. Further, the benefit of PSA screening was diminished by loss of quality-adjusted life-years owing to post-diagnosis long-term effects [10].

The role of treatment, once diagnosed, has also been questioned. The Scandinavian Prostate Cancer Group-4 (SPCG-4) and the Prostate Cancer Intervention Versus Observation Trial (PIVOT) randomised controlled trials of radical prostatectomy compared to watchful waiting have shown that the benefit of treatment in terms of improved overall survival was restricted to those men with higher risk – clinically significant – prostate cancer [11–14].

In order to minimise over-diagnosis and over-treatment, the optimal diagnostic pathway should be able to discriminate reliably between men with and without clinically significant cancer.

## 2 The current diagnostic pathway

Men deemed at risk of prostate cancer are those with an elevated PSA level, an abnormal digital rectal examination, a positive family history of prostate cancer or a specific ethnic profile [15]. Once a risk factor is identified, patients are advised to have a trans-rectal ultrasound (TRUS) guided biopsy. Annually, between 59,000 and 80,000 men have a TRUS-biopsy in the UK and about one million in the USA [16]. TRUS-biopsy can be problematic in that the ultrasound is used to identify the prostate itself and is unable to identify suspicious lesions with any degree of accuracy; this results in biopsies being taken blindly throughout the prostate. This is in contrast to the approach taken in most other solid organ cancers where the lesion is identified, usually on imaging, in order to direct biopsies to the area of suspicion. The random and systematic errors in diagnosis which are inherent in TRUS-biopsies lead to a number of problems.

### 2.1 TRUS biopsies over-diagnose clinically insignificant prostate cancer

**Fig. 1:**
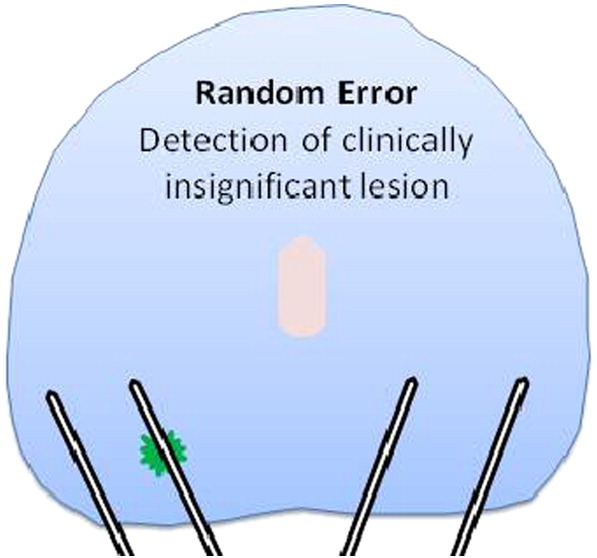
Over-detection of insignificant prostate cancer resulting from TRUS-guided biopsies.

A man who undergoes TRUS-biopsy has a 1 in 4 chance of being diagnosed with prostate cancer [17,18]. This compares with a 6–8% lifetime risk of having prostate cancer that will impact life expectancy. The over-detection of these small low-grade lesions is due in part to the random deployment of the needles [17–19] (Fig. 1).

### 2.2 TRUS-guided biopsies miss clinically significant cancers

**Fig. 2:**
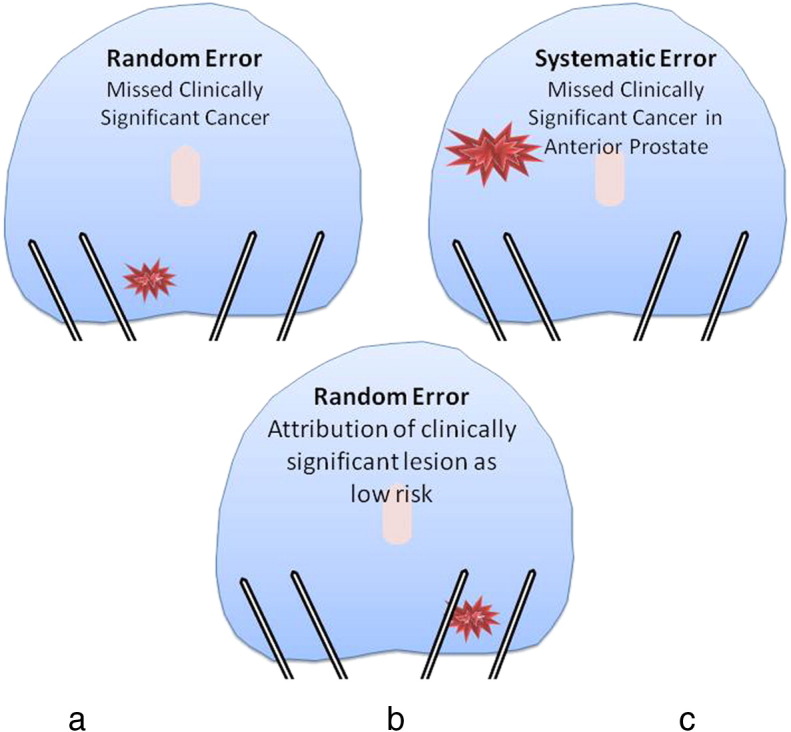
Under-detection of clinically significant prostate cancer resulting from TRUS-guided biopsies.

They have an estimated false negative rate of 30%–45% [19,20]. The clinician takes 10–12 biopsies in a manner that attempts to obtain representative tissue from the peripheral zone (Fig. 2a). However, this systematic error leads to significant cancer being missed in the peripheral zone. Further, several parts of the prostate are systematically under-sampled. First, the anterior gland is missed as a result of its greater distance from the rectum (Fig. 2b). Second, areas in the midline are under-sampled due to efforts to avoid the urethra. Third, the prostate apex is often difficult to access by the transrectal route [21–24].

### 2.3 TRUS-biopsies can misrepresent the true burden of cancer

The random sampling error (Fig. 2c) can mean that a biopsy does not hit the cancer lesion through its greatest diameter leading to either or both the size or grade of cancer being underestimated [25] (Fig. 2c). Up to half of men deemed low risk on TRUS-biopsies can have higher burden or grade, or both, when a more accurate biopsy test is applied [22–24,26–28]. As a result of the poor risk attribution, many men with true low risk disease cannot be sure of their risk attribution so they and their physicians often choose radical therapies from which they derive little to no survival benefit [11].

### 2.4 TRUS-biopsy has harms

It is associated with a number of complications, the most important being urinary tract infection (1–8%) that can result in life-threatening sepsis (1–4%). Haematuria (50%), haematospermia (30%), pain/discomfort (most), dysuria (most) and urinary retention (1%) can also be expected [29–32].

## 3 The proposed new diagnostic pathway

**Fig. 3:**
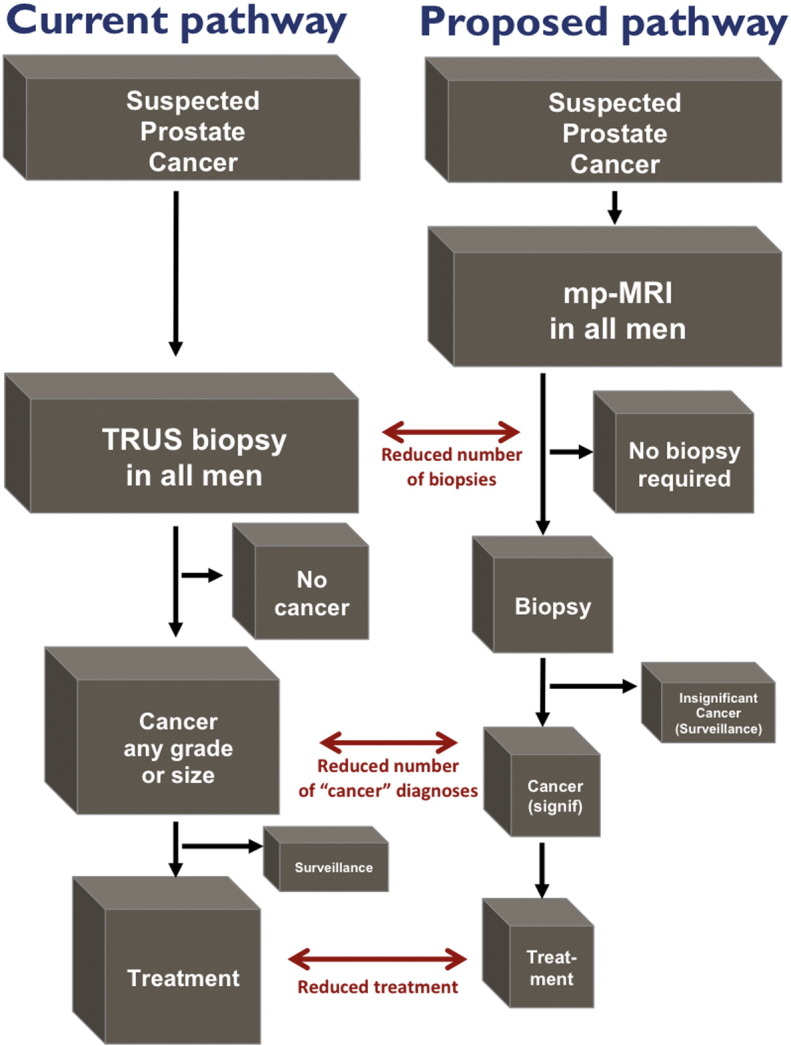
Current and proposed diagnostic pathways should MP-MRI prove favourable.

A novel pathway (Fig. 3) in which imaging is used as a triage test [33] for men at risk of prostate cancer might enable physicians to decide if the patient should or should not have a prostate biopsy, whilst those patients with a visible lesion could have a targeted biopsy.

This proposed new pathway might offer several advantages. Should the results of the trial be in favour of MP-MRI, we hypothesise three corrections in the current pathway that might occur. First, over-diagnosis might be reduced because fewer clinically insignificant prostate cancers will be detected by chance as only patients with positive MRIs will undergo a biopsy. Second, as a result there might be less over-treatment. Third, the detection of clinically significant prostate cancers might be improved. Fourth, there might be better treatment allocation to active surveillance or radical therapies due to more representative biopsy sampling and better risk stratification. Fifth, complications might be reduced if fewer men are biopsied [34,35].

At present multi-parametric MRI (MP-MRI) appears to have the desired attributes of a test that could be used in the prostate cancer diagnostic pathway [36]. The evidence base available at the time of conceiving PROMIS suggested that MP-MRI can achieve both a sensitivity and specificity between 70–90% for the detection of clinically significant prostate cancer [37]. However, a systematic review of the literature [38] found the quality of the initial studies evaluating MP-MRI to be disappointing [36]. Early reports repeatedly showed low sensitivity and specificity as well as high inter-observer variability, even when using high-resolution endorectal MRI [39–45]. Since these early reports, much has changed including an appreciation of the impact of post-biopsy changes on MR-image quality, technological improvements such as increasing magnetic field strength (from 0.5 T to 1.5 T and 3.0 T), shorter pulse sequences enabling faster image acquisition, and the introduction of functional imaging in the form of diffusion weighting (DW) and dynamic contrast-enhancement (DCE).

Multi-parametric approaches in which sequences are combined together have found an advantage for using two or three MRI sequences rather than just one. When PROMIS started, none had evaluated the clinical validity of MP-MRI in the population of interest – men at risk – against an accurate and appropriate reference standard within a prospective multi-centre study (Table 1) [46–56].

There were a number of limitations to the early studies investigating the diagnostic accuracy of MRI for prostate cancer that probably account for the limited acceptance of MRI into the prostate cancer diagnostic pathway [57]. These include:

a) *biopsy artefact*: studies mostly evaluate MRI after biopsy. This results in biopsy-related haemorrhage and inflammation, which can lead to false positives or negative results or scans which are not interpretable.
b) *Limited application*: many studies evaluated only the peripheral zone of the prostate, ignoring up to one-third of prostate cancers that lie anteriorly.
c) *Segmentation*: most studies segmented the prostate into a number of sectors for analysis in order to both achieve sufficient matched datasets. These studies classify each sector of analysis as ‘independent’. Segmentation is usually employed because the reference used is radical whole-mount prostatectomy.
d) *Inaccurate or inappropriate reference standard*: most studies used radical prostatectomy (RP), leading to selection bias as men have to test positive on a biopsy first and then choose to have surgery. Those undergoing surgery tend to have burdens of cancer that are distinct from patients choosing other treatments [58]. Further, as the prostate is usually segmented, co-registration of an image to an RP specimen is challenging because of shrinkage (10–20%), distortion, tissue loss as a result of ‘trimming’ (10%), orientation, absent perfusion and sector boundaries [59,60].

## 4 Design and methods: the PROMIS protocol

### 4.1 Design

**Fig. 4:**
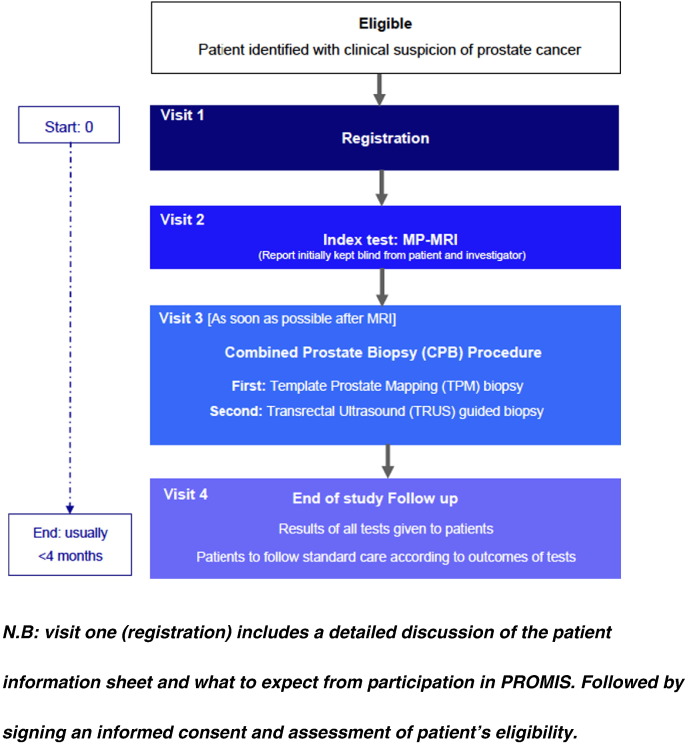
PROMIS Trial Schema. N.B.: visit one (registration) includes a detailed discussion of the patient information sheet and what to expect from participation in PROMIS. Followed by signing an informed consent and assessment of patient's eligibility.

PROMIS is a validating paired cohort study representing level 1 evidence for diagnostic studies [61]. The primary objectives of PROMIS are to assess the ability of MP-MRI to identify men who can safely avoid unnecessary biopsy and to assess the ability of the MP-MRI based pathway to improve the rate of detection of clinically significant cancer by evaluating the diagnostic accuracy of MP-MRI (the index test) against an accurate reference standard, template prostate mapping (TPM) biopsies, which can be applied to all men under evaluation. The current TRUS-biopsy (standard test) will also be evaluated against TPM. All men consenting to the study will have a MP-MRI, followed by a combined prostate biopsy procedure in which TPM biopsies will be followed by TRUS-guided biopsies. Each test will be conducted and reported independent of the other tests (Fig. 4).

PROMIS is designed to overcome shortcomings highlighted in the current literature. First, since all patients will be biopsy-naïve and undergo MP-MRI prior to any biopsy, there will be no biopsy artefact. Second, MP-MRI will be evaluated for all anatomical zones of the prostate including peripheral and transition zones. Third, the study is powered so that the primary outcome will be derived using the whole prostate as the sector of analysis rather than segmented sectors of the prostate. Fourth, PROMIS employs an accurate reference test that can be applied to all men at risk.

Furthermore, the study has been designed to ensure avoidance or minimisation of a number of biases that are inherent in the current literature. First, spectrum and selection biases will be avoided by recruiting men at risk of prostate cancer and applying all tests to all men. Second, work-up bias will be eliminated by ensuring that patients and clinicians remain blinded to all imaging test results until the biopsies have been carried out and reported. Third, reviewer/reporter bias will be avoided by ensuring that the radiologist is blinded to the reference test and the pathologist is blinded to the imaging. The radiology report in particular will be submitted prior to the biopsies. Last, incorporation bias is minimised by ensuring that TPM biopsies and TRUS-guided biopsies follow a standard accepted protocol.

### 4.2 Patient population

Men who have never had a prostate biopsy before are eligible for the study if there is a clinical suspicion that they may be harbouring prostate cancer. This essentially includes men with an elevated PSA and/or a suspicious digital rectal examination, family history of prostate cancer or an ethnic risk group. Men with a PSA above 15 ng/ml are excluded from PROMIS as physicians are unlikely to use MP-MRI as a triage test to avoid a biopsy due to the higher incidence of prostate cancer in this sub-group. The inclusion and exclusion criteria are described in Table 2. Patients who do not want to participate in PROMIS or who are ineligible are returned to the normal clinical pathway employed in the participating centre.

### 4.3 Study interventions

#### 4.3.1 The index test — multi-parametric magnetic resonance imaging

Although experts in the field generally regard the performance characteristics of MP-MRI of the prostate as promising [62], there exists professional disagreement on its accuracy and usefulness in clinical practice [35], limiting wider adoption. These concerns relate in part to the variable quality and methodology of studies that have resulted in marked variation in indication, conduct, interpretation, and reporting [46,54,60,63–66].

As a result, MP-MRI in PROMIS will be standardised to the minimal requirements advised by a European consensus meeting [67], the European Society of Uro-Radiology [68] and the British Society of Uro-Radiology guidelines [69]; this will entail acquiring T1-weighted, T2-weighted, diffusion-weighted (apparent diffusion coefficient maps and long-b scan) and dynamic gadolinium contrast-enhanced imaging using a 1.5 Tesla scanner and a pelvic phased array (Table 3).

Use of endorectal coils will be avoided, as there is no consensus on its role in minimal scanning requirements [67]. It was decided to not include magnetic resonance spectroscopy as evidence from a large prospective multicentre study at the time showed no benefit of spectroscopy for prostate cancer localization compared with T2-weighted imaging alone [70].

In order to maintain the quality of scans and ensure uniformity across all centres, optimization of the conduct of scans will be applied to all centres through a robust quality control process. This will be undertaken by a separate independent commercial sub-contractor (Ixico Ltd, UK) selected through an open competition compliant with the European Union guidelines on the tender process. Scans deemed of insufficient quality by the commercial partner or the reporting radiologist will be repeated.

##### 4.3.1.1 Standardization of MRI reporting

**Fig. 5:**
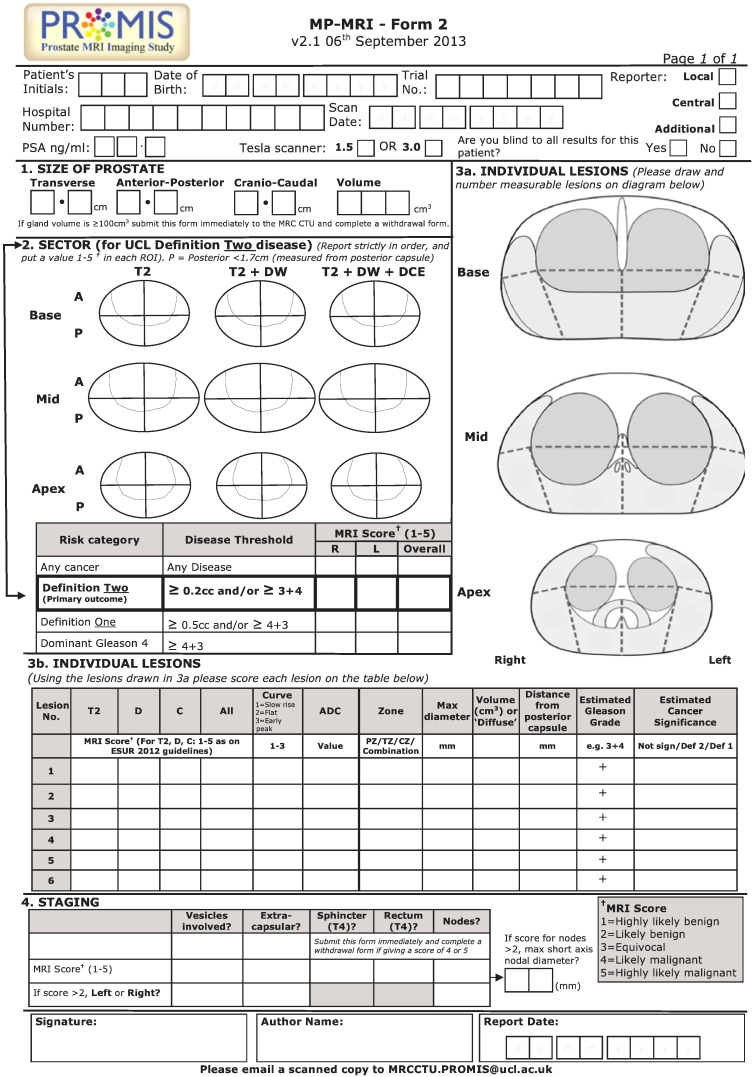
MP-MRI reporting form.

In order to avoid variation in method of interpretation, a standardised operating procedure for MP-MRI reporting has been adopted in line with the recommendations of the European consensus meeting and the European Society of Uro-Radiology prostate MRI guidelines [68]. All radiologists will undergo training and standardisation of reporting by the lead radiologist centrally prior to reporting within the trial. The actual reporting will require all radiologists being provided with the same clinical details including PSA, DRE findings and any other risk factors. Images will be reported in sequence so that T2-weighted images will be reported first, T2-weighted and diffusion-weighted together and then a third report issued for T2-weighted with diffusion and dynamic contrast enhanced scans together. A separate report will be produced for each combination of sequences in order to secondarily investigate whether both diffusion-weighted and DCE are both required. As DCE requires contrast agent (with its need for intravenous access, medical supervision and contrast-related risks) and an additional 10–15 min of scan time there is considerable merit in determining whether this additional resource and cost is necessary (Fig. 5).

A 1 to 5 Likert scoring system [67,68,71] will be used to indicate probability of cancer (1 — highly likely to be benign, 2 — likely to be benign, 3 — equivocal, 4 — likely to be malignant and 5 — highly likely to be malignant) with the prostate divided into 12 regions of interest (ROI) and each region scored from 1 to 5. Further, each lesion will be identified and scored on the 1 to 5 scale separately and the longest axial diameter, lesion volume, ADC value and contrast enhancement curve type will be recorded [72–75]. An overall 1 to 5 score of the whole prostate will be recorded for each level of cancer burden that the radiologist thinks might be present. This will be ‘all cancer’, ‘definition 1 clinically significant cancer’ and ‘definition 2 clinically significant cancer’ (see below).

With respect to the primary outcome, an overall score of 3 or more will be used to indicate the possible presence of clinically significant cancer (i.e., a positive MP-MRI score). This reflects the level at which further tests (e.g., biopsy) would be considered if MP-MRI were to be introduced into the diagnostic pathway in the future.

##### 4.3.1.2 Assessing for inter-observer variability and quality control and assessment

In order to establish if a diagnostic test can improve or change the diagnostic pathway in prostate cancer, it must be assessed for intra- and inter-observer variability. Thus, a subset of scans will be reported by another experienced central radiologist. A subset of scans will also be reported by the same reporter again at a different time-point to assess intra-observer variability.

In order to make sure that the result of the MP-MRI does not influence the conduct of the biopsy, the results of the MP-MRI will not be revealed to either the men having the biopsies or to the clinicians undertaking the biopsies until after the results of the TRUS-biopsy and TPM biopsies are available (with the exceptions for un-blinding given below). This blinding is necessary to prevent the results of the MP-MRI influencing whether men are biopsied and if they are, how the biopsies are conducted.

For safety purposes, the results of the MP-MRI can be un-blinded by the radiologist if the MP-MRI reveals an enlarged prostate > 100 ml in volume or there is evidence of T4 prostate cancer or involved lymph nodes or colorectal/bladder invasion. The presence of other cancers such as bladder or colorectal cancers will also be a criterion for withdrawal. This information will be provided to the treating clinician for appropriate clinical decision making.

#### 4.3.2 The standard test — transrectal ultrasound-guided biopsy

TRUS-biopsy of the prostate is to be performed after TPM biopsies, under the same general/spinal anaesthetic. This helps ensure that results are obtained for the reference test in an optimal fashion in a biopsy naïve gland that has not undergone swelling and distortion. It also theoretically minimises the risk of infection as the potential for faecal contamination is restricted to the end of the procedure. The surgeon performing the biopsy procedure will be blind to the MRI results so no targeting of suspicious areas will occur. TRUS-guided biopsies incorporate 10–12 core biopsies taken as per international guidelines [76]. Each core will be identified and potted separately. The TPM biopsies and TRUS-guided biopsy sets from a particular patient will be sent to different pathologists to minimise review and work-up biases.

#### 4.3.3 Comparator

##### 4.3.3.1 Transperineal Template Prostate Mapping biopsies

**Fig. 6:**
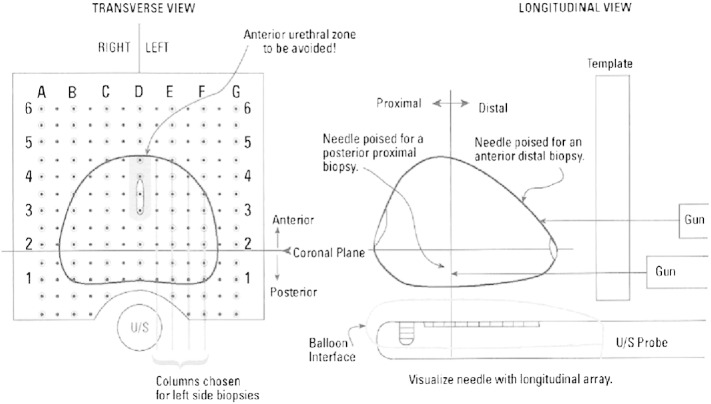
Illustration of how Transperineal Template Prostate Mapping biopsies are conducted.

Transperineal Template Prostate Mapping (TPM) biopsies (Fig. 6) has been selected as the reference test; when using 5 mm-sampling it meets the required specification as a reference test for our defined population [24,26,77–80].

TPM biopsies produce a histological map of the entire prostate in 3-dimensions with an estimated sensitivity and negative predictive value (NPV) in the order of 95% for clinically significant cancers when assessed against radical prostatectomy [21,24]. TPM biopsies have a similar side-effect profile to that of TRUS-biopsy with three important differences. First, they carry a significantly lower risk of urosepsis (< 0.5%) – the most serious complication of TRUS-biopsy – as the needles do not traverse the rectal mucosa. Second, TPM biopsies confer a higher risk of self-limiting failure to void urine (5–10%) as a result of greater gland swelling [78,81,82] compared to 1–2% risk associated with TRUS-biopsy. Third, TPM biopsies require a general/spinal anaesthetic. The accuracy of TPM biopsies is high and has been recently validated against radical whole-mount specimens [83]. We chose to combine TPM biopsies with TRUS-biopsies under the same general/spinal anaesthetic in order to reduce patient burden (from two visits and two procedures to one visit) and minimise drop-out of patients between tests. Training will be provided to all centres to conduct TPM biopsies according to the PROMIS protocol although all centres have been selected for their prior experience in carrying out TPM biopsies.

##### 4.3.3.2 Side effect profile of a combined TPM and TRUS-biopsy procedure

The expected side-effects of combining both procedures is detailed in the patient information sheet which is discussed with all patients prior to registration (Table 4). Our rate of serious adverse events is monitored by an independent trial steering committee on a weekly basis.

### 4.4 The target condition for detection — defining clinically significant prostate cancer

**Fig. 7:**
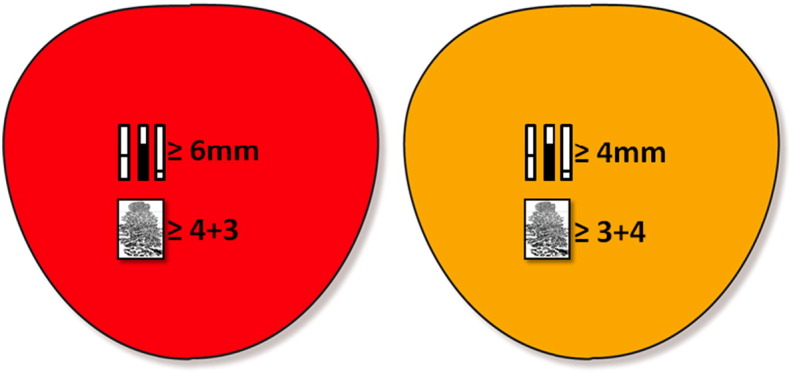
Definitions of clinical significance on TTPM-biopsy. Red signifies UCL definition 1 against which the primary outcome will be validated. Yellow signifies UCL definition 2 and is a secondary outcome.

Despite the high accuracy of TPM biopsies in detecting clinically significant disease there is no widespread agreement on the criteria to define clinically significant prostate cancer on TRUS-biopsies or TPM biopsies. Whilst some definitions exist, such as the Epstein criteria [84], they have been developed for TRUS-biopsy; indeed, if these were applied to TPM biopsies the prevalence of intermediate and high risk disease would be artificially inflated given the different sampling densities used [28,85]. As a consequence, the best evidence to select the risk classification for analysing TPM biopsy results has been derived by a simulation study using TPM-biopsies with a 5 mm sampling density. By combining maximum cancer core length and Gleason score, this simulation study stratified patients into three groups: low risk, intermediate risk and high risk (Fig. 7). The target definition for clinically significant disease on TPM biopsies for the primary outcome will be set at a maximum cancer core involvement >/= 6 mm and/or Gleason >/= 4 + 3 (UCL definition 1). The cancer core length in particular relates to an area of cancer on TPM biopsies that approximates to a lesion volume of >/= 0.5 ml [17]. We chose this target condition as the primary outcome on the basis that few physicians would disagree that any man having this burden of cancer would require treatment. A further threshold for clinically significant disease will also be used (cancer core length involvement >/= 4 mm and/or Gleason >/= 3 + 4) (UCL definition 2).

### 4.5 Outcomes

#### 4.5.1 Primary outcomes

The primary outcomes in this trial are of fundamental importance to decisions regarding the future use of MP-MRI in the diagnostic pathway for the prostate cancer. First, they include the proportion of men who could safely avoid biopsy as determined by specificity and negative predictive values (NPVs) for clinically significant cancer. Second, the proportion of men correctly identified by MP-MRI to have clinically significant prostate cancer as determined by sensitivity and positive predictive values (PPV). For the primary outcomes, UCL definition one criteria will be used to set the target definition of clinically significant prostate cancer on TPM biopsy and a score of 3 or more on MP-MRI will be used to define a positive index test. Further, the accuracy of TRUS-biopsy will also be reported in terms of sensitivity, specificity, NPV, and PPV as listed in the section below. In addition, a head-to-head comparison of the accuracy of MP-MRI versus TRUS-guided biopsy (current standard) will be performed. All primary and secondary outcomes are presented in Table 5.

### 4.6 Translational objectives

PROMIS is ideal for assessing the utility of biomarkers (from urine and blood) to identify men with clinically significant prostate cancer. This is the first time that a broad spectrum of men at risk will be evaluated using an optimal biopsy technique that accurately characterises the presence, size and grade of prostate cancer. We will collect, process and store a comprehensive bank of tissue samples (serum, plasma, germ-line DNA, urine) from men prior to biopsy, to analyse urinary and serum biomarkers with respect to the detection of clinically significant prostate cancer on TPM biopsy.

### 4.7 Trial conduct

The study has been set up to run in two stages: the pilot phase, followed by the main phase. The pilot study has already completed and recruited 50 patients over one year to allow testing of safety and recruitment. At the end of the pilot in May 2013, few safety concerns emerged and the Trial Steering Committee recommended continuation of the study into the main phase [86].

If the combination of TRUS-biopsy and TPM biopsies leads to more than a 4% risk of sepsis at any time, there could be cause for concern and a requirement for modification of the study design. If deemed appropriate, recruitment to the study will be suspended until any safety concerns have been resolved.

### 4.8 Long-term follow-up through linkage

The long-term outcomes of the PROMIS cohort will be of interest and contribute to our understanding of the epidemiology of prostate cancer. Men who specifically consent to long-term data collection will be flagged and followed up using the Office for National Statistics and NHS databases. For example, linkage to Hospital Episode Statistics (HES) may give valuable information on further diagnoses, treatments and outcomes beyond the timeframe of the study for future analyses. Consenting men may additionally be contacted in the future to assess their willingness to respond to questionnaires. This allows the potential for research that would complement the planned long-term follow-up in terms of health status, for example picking up future biopsies not included in HES, and allows assessment of quality of life.

### 4.9 Statistical considerations

#### 4.9.1 Sample size

Power calculations were performed in relation to: (1) precision around the estimates for the accuracy of MP-MRI in terms of the joint primary outcomes of specificity and sensitivity, and (2) a head-to-head comparison of the MP-MRI versus TRUS. The largest sample size from (1) and (2) was 714 (as detailed below) and this was taken as the maximum number of men required to have all 3 tests (MP-MRI, TRUS biopsy and TTPM-biopsies).

#### 4.9.2 Prevalence of clinically significant cancer

**Fig. 8:**
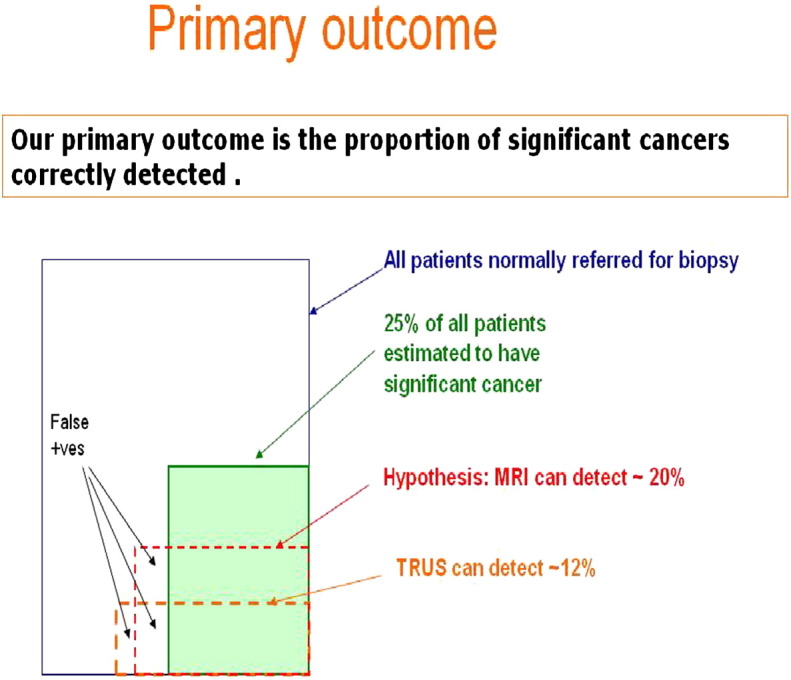
Illustration summarizing some of the assumptions made in determining sample size calculations for the primary outcome.

For all calculations we have assumed [19,20,28,87] that 15% of the study population will have clinically significant prostate cancer as detected by the reference standard according to UCL definition one and 25% will have clinically significant prostate cancer as detected by the reference standard according to UCL definition two. These estimates act as inflation factors for the total number of men required for the study. All calculations are based on 90% power and 5% significance (2-sided). The specified estimates of sensitivity and specificity are considered realistic based on current unpublished and published literature [88,89] (Fig. 8).

#### 4.9.3 Specificity of MP-MRI

Assuming a specificity of 77%, in order to demonstrate that the lower 95% confidence interval of specificity is at least 70% or greater, we would require 407 cases of negative or clinically insignificant prostate cancer. This is equivalent to a total of 479 men for UCL definition one and 543 men for UCL definition two.

#### 4.9.4 Sensitivity of MP-MRI

Assuming a sensitivity of 75%, in order to demonstrate that the lower 95% confidence interval of sensitivity is at least 60% or greater, we would require 97 cases of clinically significant prostate cancer. This is equivalent to a total of *647* men for UCL definition one and *388* men for UCL definition two. Since the number of men without clinically significant prostate cancer will be much higher than the number with, the precision for estimating specificity and NPV is much greater.

#### 4.9.5 MP-MRI versus TRUS-biopsy

We have assumed that TRUS-biopsy detects 48% of clinically significant prostate cancer [28,90] and MP-MRI will detect at least 70% (conservative estimates). Using McNemar's test for paired binary observations [91], in order to show an absolute increase in the proportion of clinically significant cancers detected of at least 22% (from 48% to 70%) with a power of 90% and a 2-sided alpha of 5%, a total of 107 cases are required. This is equivalent to a total study population of 714 men for UCL definition one and 428 men for UCL definition two.

#### 4.9.6 Cost effectiveness analyses

A model will be populated from the study as well as a review of secondary sources of epidemiological, clinical and economic evidence together with appropriately elicited expert opinion [92]. The use of probabilistic sensitivity analysis, value of information methods and scenario analysis [93] will quantify the uncertainty associated with identifying the most cost-effective diagnostic strategy, the costs of that uncertainty (in health and resource terms) and the key uncertainties to resolve with further research. This will inform the inputs into the main economic model. This cost-effectiveness model will seek to quantify the long-term implication of changes to the diagnostic classification of prostate cancer that result from adoption of alternative diagnostic pathways within the NHS. The implications will relate to the health effects (in terms of quality adjusted life expectancy) and NHS costs of a given diagnostic pathway placing patients into each of the four groups: 1. MRI test positive, clinically significant disease; 2. MRI test negative, clinically significant disease; 3. MRI test positive, clinically insignificant disease; and 4. MRI test negative, clinically insignificant disease. By altering the likelihood of a man falling into any one of these groups, the value of MP-MRI will be assessed by the changes in average outcomes experienced by men and the costs that result. The model will also include the implications of a positive result in the index test concurrent with a negative result in the current standard as well as accounting for the side effect profile of different diagnostic pathways. Structurally, the model will consist of a diagnostic element that will model the probabilities of a given patient falling into each of the diagnostic groups above, and a prognostic element that will estimate the long term implications for health and costs. The specific details of model structure will be informed by a review of existing prostate cancer models including those relating to screening, diagnosis and treatment. In general terms the modelling will adhere to the methods advocated to inform guidance by the UK National Institute for Health and Clinical Excellence [94].

We will also collect data on the costs of tests and the management of adverse events, and the health-related quality of life (HRQL) implications of any adverse events experienced with tests. The latter will be assessed using the EQ-5D instrument as part of the main clinical study. This is a widely used generic measure of HRQL which can be used to derive quality adjusted life years (QALYs) [95]. Ultimately, this work will provide an assessment of the implications of any change that the use of MP-MRI has on under-detection and over-detection. These implications will be in terms of expected quality adjusted survival duration and long-term health service costs. This will allow the value for money of MP-MRI in this context to be assessed using the same metrics employed to evaluate therapeutic technologies by organisations such as NICE.

### 4.10 Ethical considerations

The study abides by the principles of the Declaration of Helsinki and the UK Research Governance Framework version 2 and received UK Research Ethics Committee approval on 16th March 2011 by the NRES Committee London—Hampstead. PROMIS is published on clinical trials.gov [96]

## 5 Discussion and limitations

The PROMIS protocol has some potential limitations. First, the thresholds we have used for clinically significant disease are open to debate as no universally accepted definition exists. It is widely accepted that some prostate cancer lesions are clinically significant and others are not [97,99]. Volume thresholds of 0.5 ml and 1.3 ml for low grade Gleason 6 lesions have been supported by recent data from the European Prostate Cancer Screening trial [98]. There are even some calls for such lesions to be re-designated as something other than malignant, such is their indolent behaviour [97,99,100]. However, we recognize that there is legitimate professional disagreement on what constitutes clinically significant prostate cancer, so we decided to reflect this by using other disease burden thresholds to define the target condition on the reference test for the purpose of validating mpMRI.

Second, TPM biopsies may not be as accurate as whole-mount prostatectomy, but a number of studies point to its accuracy being sufficiently high to use as a reference test for the specific population we will recruit. Indeed, for men with no cancer diagnosis or for those not choosing surgery, it is the best available reference standard.

Third, the sequence of TPM biopsy first followed by TRUS-guided biopsy might compromise the standard test. However, the decision for this sequence was one primarily based around safety — inoculation of bacteria by TRUS-guided biopsy into the gland followed by numerous TPM biopsies may theoretically increase sepsis risk. We also wanted to ensure that the reference test was not compromised by swelling caused by the TRUS-guided biopsy if the latter was performed first. Further, because TRUS-guided biopsies were being performed under general/spinal anaesthetic with rectal cleansing performed using 2% chlorhexidine solution might actually be better than standard care in terms of sepsis and test accuracy.

## 6 Conclusion

PROMIS will determine whether introduction of MP-MRI prior to biopsy can safely allow men to avoid a biopsy and its associated harms. It will also determine whether MP-MRI can better identify men with clinically significant disease that requires a biopsy to confirm diagnosis using accurate targeting to the lesion. The evidence produced by PROMIS will aid current research interest investigating the possibility of directing biopsies only to the suspicious areas on MRI without deploying systemic biopsies.

Much research has focused on developing and validating novel imaging and tissue biomarkers for early detection of clinically significant prostate cancer. These programmes of research have used TRUS-biopsy as the reference test with any volume, grade and risk of cancer taken as a ‘positive’. PROMIS aims to overcome the problems of TRUS-biopsy as a reference test by using TPM biopsies that have a very high degree of accuracy and can be applied to all eligible men. It therefore represents an opportunity to develop and validate numerous imaging and tissue biomarkers in their performance characteristics to discriminate between men at risk who have absence of clinically significant cancer and those men who have clinically significant cancer.

## Trial status

Currently PROMIS is open for recruitment across 11 centres in the United Kingdom with 3 more undergoing site setup (Appendix 1). We are on target to conclude our recruitment. Recruitment commenced in May 2012 and is expected to come to an end in October 2015.

## Funding

National Institute of Health Research — Health Technology Assessment: Project Number 09/22/67.
  **Department of Health Disclaimer**:
  The views and opinions expressed therein are those of the authors and do not necessarily reflect those of the health technology assessment programme, NIHR, NHS or the Department of Health.
Prostate Cancer UK: funding blood and urine collection and processing for the translational aspect of PROMIS (PROMIS-T).

## Author contributions

PROMIS has been fortunate to receive input and advice from a wide range of experts in their respective fields detailed in Appendix 1.

**Study concept and initial design**: Parker, Emberton, Ahmed.

**Study design and statistical analysis**: Kaplan, Brown, Gaibe.

**Acquisition of data and Data analysis and interpretation**: Hindley, El-Shater Bosaily.

## Figures and Tables

**Table:** 

**Trial sponsor:**	University College London (UCL)
**Trial coordination:**	Medical Research Council Clinical Trial Unit (MRC CTU) at UCL
**Funders:**	National Institute for Health Research (NIHR) Health Technology Assessment (HTA) & Prostate Cancer UK

**Trial Management Group:**	
Professor Mark Emberton (Chief Investigator)	UCLH (Urologist)
Mr Hashim Ahmed (Co-chief Investigator)	UCLH (Urologist)
Dr Ahmed El-Shater Bosaily	UCLH (Clinical Research Fellow)
Dr Alex Kirkham	UCLH (Radiologist)
Dr Alex Freeman	UCLH (Pathologist)
Dr Charles Jameson	UCLH (Pathologist)
Mr Richard Hindley	Basingstoke (Urologist)
Dr Christopher Parker	Royal Marsden (Translational Research)
Professor Colin Cooper	Royal Marsden (Translational Research)
Robert Oldroyd	Patient representative
Professor Richard Kaplan	MRC CTU
(Programme Lead/Oncologist)	
Dr Louise Brown	MRC CTU
(Project Lead/statistician)	
Dr Rhian Gabe	University of York (Statistician)
Dr Yolanda Collaco-Moraes	MRC CTU (Clinical Operations Manager)
Cybil Adusei, Katie Ward	MRC CTU (Trial Manager)
Sophie Stewart, Katie Thompson	
Claire Mulrenan, Hannah Gardner	MRC CTU (Data Managers)
Carlos Diaz-Montana	MRC CTU (Data programmer)
Dr Chris Coyle	MRC CTU (Clinical Fellow)
Professor Mark Sculpher	University of York (Health Economics)
Dr Rita Faria	University of York (Health Economics)

**Trial Steering Committee (also act as Data Monitoring Committee):**	
Dr David Guthrie (Chair)	Derbyshire Royal Infirmary (Oncologist)
Professor John Chester	University of Cardiff (Oncologist)
Professor Richard Cowan	Christie Hospital Manchester (Oncologist)
Professor Michael Jewitt	University of Toronto (Urologist)
**Participating centres:**	
*University College London Hospital*: H. Ahmed (PI), J. Coe, A. El-Shater Bosaily, M. Emberton, A. Freeman, M. Hung, C. Jameson, A. Kirkham, S. Punwani, R. Scott.	
*Basingstoke General Hospital*: Richard Hindley (PI), A. Edwards, H. El-Mahallawi, D. Peppercorn, J. Smith, A. Thrower.	
*Imperial College London*: M. Winkler (PI), K. Ansu, T. Barwick, S. Edwards, L. Honeyfield, N. Qazi, B. Statton, V. Stewart, E. Temple.	
*Musgrove Park Hospital*: N. Burns-Cox (PI), P. Burn, K. Gordon, H. Routley, A. Maccormick, D. Paterson.	
*Maidstone and Tunbridge Wells Hospital*: A. Henderson (PI), E. Bernsten, R. Casey, D. Day, S. Ghosh, J. James, P.J. McMillan, G. Russell.	
*Southmead Hospital, Bristol*: R. Persad (PI), J. Ash-Miles, M. Elmahdy, S. Pandian and C. Shiridzinomwa, M. Sohail, A. Treasure.	
*Whittington Hospital, London*: M. Ghei (PI), V. Conteh, L. Harbin, R. Katz, J. Kumaradevan, A. Trinidade, A. Verjee.	
*Southampton General Hospital*: T. Dudderidge (PI), J. Smart	
*Sheffield University Hospitals*: D. Rosario (PI), J. Catto, F. Selem	
*Wrexham Maelor Hospital, Betsi Cadwaladr University Health Board, Wrexham, Wales*: I. Shergill (PI), S. Agarwal	
*Frimley Park Hospital*: S. Bott (PI), H. Evans	

From Ahmed HU et al., J Urol, 2011; 186(2):458-64.

**Table 1:** Sensitivity and specificity of MRI parameters as reported in the literature.

Parameter	Number (mean)	Sensitivity	Specificity
T2	12–320 (97)	37–96%	21–67%
DW	11–95 (42)	57–90%	79–88%
DCE	23–54 (41)	71–87%	61–89%

**Table 2:** Patient inclusion and exclusion criteria.

Patient inclusion criteria
Men at least 18 years or over at risk of prostate cancer who have been advised to have a prostate biopsy
Serum PSA ≤ 15 ng/ml within the previous 3 months
Suspected stage ≤ T2 on rectal examination (organ confined)
Fit for general/spinal anaesthesia
Fit to undergo all protocol procedures including a transrectal ultrasound
Signed informed consent

Patient exclusion criteria

Treated using 5-alpha-reductase inhibitors at time of registration or during the prior 6 months
Previous history of prostate biopsy, prostate surgery or treatment for prostate cancer (interventions for benign prostatic hyperplasia/bladder outflow obstruction is acceptable)
Evidence of a urinary tract infection or history of acute prostatitis within the last 3 months
Contraindication to MRI (e.g., claustrophobia, pacemaker, estimated GFR ≤ 50)
Any other medical condition precluding procedures described in the protocol
Previous history of hip replacement surgery, metallic hip replacement or extensive pelvic orthopaedic metal work.

**Table 3:** Standard operating procedure for MRI parameters for all centres to follow.

	TR	TE	Flip angle/degrees	Plane	Slice thickness (gap)	Matrix size	Field of view/mm	Time for scan
T2 TSE	5170	92	180	Axial, coronal, sagittal	3 mm (10% gap)	256 × 256	180 × 180	3 min 54 s (ax)
VIBE at multiple flip angles for T1 calculation (optional)								Will be included in the Phoenix file
VIBE fat sat	5.61	2.52	15	Axial	3 mm	192 × 192	260 × 260	Continue for at least 5 min 30 s after contrast
Diffusion (b values: 0, 150, 500, 1000)	2200	Min (< 98)		Axial	5 mm	172 × 172	260 × 260	5 min 44 s (16 averages)
Diffusion (b = 1400)	2200	Min (< 98)		Axial	5 mm	172 × 172	320 × 320	3 min 39 s (32 averages)

**Table 4:** Combined prostate biopsy procedure side effect profile as stated in the patient information sheet and consent documentation.

Side effect	Procedure	
	TRUS alone (standard care)	Combined biopsy: TPM + TRUS (in the PROMIS study)
Pain/discomfort	Almost all men experience temporary discomfort in the rectum	Almost all men experience temporary discomfort in the rectum
Burning when passing urine	Almost all men	Almost all men
Bloody urine	1 in 2 men (self-resolving, 2–3 days)	Almost all men (self-resolving, 2–3 days)
Bloody sperm	3 in 10 men (2–3 months to resolve)	Almost all men (lasting up to 3 months)
Poor erections	3 in 10 men (self-resolving after 6–8 weeks). Rarely, tablets may be needed to help the erections improve.	Almost all men (self-resolving after 6–8 weeks). Rarely, tablets may be needed to help the erections improve.
Infection of skin or urine	1–8 in 100 men	1–8 in 100 men
Infection of skin or urine requiring admission and intravenous antibiotics	Between 1–4 in 100 men	Between 1–4 in 100 men
Difficulty passing urine	1 in 100 men	1–3 in 20 men
Bruising of skin	None	Almost all men
Bruising spread to scrotum	None	Between 1 in 20 to 1 in 10 men

**Table 5:** Primary and secondary outcomes for the PROMIS trial.

Primary outcomes:
Proportion of men who could safely avoid a biopsy as determined by specificity and negative predictive values (NPV), based on definition one of clinical significance as assessed by TPM.
Proportion of men correctly identified by MP-MRI to have clinically significant prostate cancer as determined by sensitivity and positive predictive value, based on definition one of clinical significance as assessed by TPM.

Secondary outcomes:

The proportion of men who could safely avoid biopsy, given that they do not have definition two prostate cancer as assessed by TPM.
The proportion of men testing positive on MP-MRI out of those with DEFINITION TWO prostate cancer assessed by TPM.
Performance characteristics of TRUS versus TPM (sensitivity, specificity, NPV, PPV) according to definitions one and two
Evaluation of the optimal combination of MP-MRI functional parameters (T2, DW, DCE) to detect or rule-out clinically significant prostate cancer.
Intra-observer variability in the reporting of MP-MRI.
Inter-observer variability in the reporting of MP-MRI.
Evaluation of socio-demographic, clinical, imaging and radiological variables in relation to the detection of clinically significant prostate cancer.
Patients' health-related quality of life using the EQ-5D instrument.
Resource use and costs for further economic evaluation (see section on Cost-effectiveness analyses).
